# Grey Level and Noise Evaluation of a Foveon X3 Image Sensor: A Statistical and Experimental Approach

**DOI:** 10.3390/s120810339

**Published:** 2012-07-31

**Authors:** Gabriel Riutort-Mayol, Ángel Marqués-Mateu, Ana Elena Seguí, José Luis Lerma

**Affiliations:** Department of Cartographic Engineering, Geodesy and Photogrammetry, Universitat Politècnica de València, Valencia 46022, Spain; E-Mails: gabriuma@upv.es (G.R.-M.); amarques@upv.es (A.M.-M.); ansegil@upv.es (A.E.S.)

**Keywords:** noise, radiometry, grey level values, digital image, linear model (LM), design of experiments (DoE), photon transfer method (PTM)

## Abstract

Radiometric values on digital imagery are affected by several sources of uncertainty. A practical, comprehensive and flexible procedure to analyze the radiometric values and the uncertainty effects due to the camera sensor system is described in this paper. The procedure is performed on the grey level output signal using image raw units with digital numbers ranging from 0 to 2^12^-1. The procedure is entirely based on statistical and experimental techniques. Design of Experiments (DoE) for Linear Models (LM) are derived to analyze the radiometric values and estimate the uncertainty. The presented linear model integrates all the individual sensor noise sources in one global component and characterizes the radiometric values and the uncertainty effects according to the influential factors such as the scene reflectance, wavelength range and time. The experiments are carried out under laboratory conditions to minimize the rest of uncertainty sources that might affect the radiometric values. It is confirmed the flexibility of the procedure to model and characterize the radiometric values, as well as to determine the behaviour of two phenomena when dealing with image sensors: the noise of a single image and the stability (trend and noise) of a sequence of images.

## 1. Introduction

Photogrammetric applications require calibrated sensors, not only in geometry but in radiometry and color. Sensor evaluation in operational and laboratory conditions is essential to characterize the many factors affecting the radiometric and geometric properties and to find out the limitations of the systems. Geometric processing applications have reached a high maturity level, but the radiometric processing applications are still in its infancy [1,2].

The characterization of the radiometric values of a sensor is a preliminary stage of calibration of photogrammetric sensors [3,4]. The complete radiometric process involves both absolute and relative calibration. Absolute calibration determines the parameters that are needed to transform grey level values into units of radiance (W/(m^2^·sr·nm)). Relative calibration normalizes the output of the sensor so that a uniform response is obtained in the entire image area when the focal plane of the sensor is irradiated with a uniform radiance field.

The characterization concerns the knowledge of the factors and the quantification of the effects on radiometric values of a sensor [5]. In this paper, we refer to the characterization of the radiometric values (grey level values) to explain the variability of the grey level values in practical imaging applications. Experimental techniques will be used to analyze the variety of factors that affect the imaging process.

The radiometric response is an observational process that encompasses different responses from different sources such as electromagnetic radiation, optical system, electronics and object scene. The uncertainty of radiometric values fundamentally limits the distinguishable content in an image and can significantly reduce the robustness of an image processing application. It is important to analyze and characterize the uncertainty effects of the radiometric values.

Table 1 outlines the most important uncertainty components, together with their sources and effects on the radiometric values. The noise component is fundamentally caused by the behaviour of the camera sensor system, by the integration time, by the bidirectional reflectance factor and by additional imaging operators. The noise due to the sensor system is always present in any image and is due the behaviour of the photo-sensitive and electronic devices. It is commonly referred as *temporal noise*, widely studied in the literature and well understood [6,7].

The spatial variability component is caused by the sensor system (spatial non-uniformities) [6,8,9], by the optical system (vignetting effect) [10], sensors manufacturing imperfections (gradual variations) [11], by non-uniform illumination [12] and by geometric shooting conditions. The temporal variability component is caused by the sensor system (temporal non-uniformity and trend effects) and by scene changes in illumination or reflectance [12].

In this paper we analyze the noise component due to the sensor system, the spatial non-uniformity effect of a single image, and the temporal non-uniformity and trend effects of a sequence of images (Table 1). The rest of the uncertainty sources are minimized during the experimentation. In fact, the vignetting effect is negligible using an optimum optical system (lens) attached to the SLR camera. Furthermore, there is light uniformity and non-scene changes in illumination with 0°/45° lighting geometry, controlled reflectivity values and shooting conditions inside a light booth. Integration time uncertainty source was isolated after fixing constant values. Additional imaging operators such as color and gamma corrections were disabled, although some authors model them [13].

A practical and comprehensive experimental and statistical procedure, the DoE for LM, is presented in the next sections to analyze and characterize the grey level values, in particular, the linearity, the noise and the temporal trend of the sensor. It is tested on a Foveon X3 CMOS sensor featured in a Sigma SD15 digital single lens reflex (SLR) camera.

The presented new approach to evaluate image sensors adds flexibility to define parameters and relationships in the mathematical model. Furthermore, its implementation is easy and practical, and can be carried out in many laboratories, which is essential for applied disciplines such as photogrammetry and computer vision.

## 2. Background

The radiometric properties and their uncertainty effects on digital images are widely studied and well understood in the literature. There are many scientific articles evaluating these topics [6,14–17]. There exists an International Standard (ISO 15739) [18] for measuring the noise of digital cameras. Also, there exists a standard for characterization of image sensors and cameras delivered by the European Machine Vision Association, the EMVA Standard 1288 [11]. The Photon Transfer Method (PTM) describes the setup to compute both the quantum efficiency and the system gain even without the measure of the number of electrons inside the sensor [19].

The spatial non-uniformities in CMOS and CCD sensor are different, being higher in CMOS. Also, the spatial non-uniformitiy is not distributed randomly across the image in CMOS sensors. In [8] the authors analyze and decompose this effect into its pixel and column components, using spatial autoregressive functions. In this paper, we deal with the spatial non-uniformity as a random distributed spatial effect across the image, likewise in CCD image sensors, since we have developed a general analysis procedure using the same approach as in ISO and EMVA standards.

### Standards for Electronic Noise Characterization

There exist standards for electronic noise characterization of image sensors [11,18]:

- ISO 15739:2003 Photography—Electronic still-picture imaging—Noise measurements.
- European Machine Vision Association (EMVA) Standard 1288, Standard for Characterization of Image Sensors and Cameras.

Both standards agree on the definition and assignment of the noise components and are focused mainly on the noise and the spatial non-uniformity due to the sensor system (Table 1). The noise evaluation methods developed by these standards are subject to some assumptions, such as:

- Linear sensitivity (photo-response) of the sensor, *i.e.*, the radiometric response (grey level values) increases linearly with the number of photons received.
- All noise components are stationary and white with respect to time and space. The parameters describing the noise component are invariant with respect to time and space.
- Only the total quantum efficiency is wavelength dependent, *i.e.*, the effects caused by light of different wavelengths can be linearly superimposed.

If these conditions are not fulfilled, the computed parameters are meaningless [6]. These standards establish that the total noise of an image is basically composed of the photo-response noise of a sensor element (pixel) and the spatial non-uniformities of a sensor array (image). The photo-response noise is commonly called *temporal noise* because it also acts based on time (different exposures). This is basically composed of an effect due to Poisson distributed statistical fluctuations of the accumulated charge (electrons), often referred to as *shot noise*, an effect due to the *signal quantification noise* which is signal dependent and uniform-distributed between the quantification intervals. Eventually, all others noise sources such as dark current, read out and amplifier circuits can be included into one normal-distributed signal independent noise source, often referred to as *dark noise*. Due to the linear sensitivity of a sensor, these noise components are additive.

Thus, the temporal noise represents the different photo-response among pixels and also, equivalently, the different photo-response among different exposures, both under the assumption that the mean response is stable with respect to space and time, that is, the temporal noise is stationary in space and time. Consequently, the temporal noise does not consider the non-uniformity of the mean response.

PTM is based on the photo-response noise (temporal noise) with and without light to determine all the parameters characterizing completely the sensor radiometry. These parameters are: the overall system gain, the quantum efficiency, the saturation signal, the temporal dark noise, the absolute sensitivity threshold, the saturation capacity, the signal-to-noise ratio and the dynamic range.

The spatial non-uniformity is commonly called *fixed pattern noise* because it is consistent for every exposure, but randomly distributed across the sensor array. The spatial non-uniformity causes different mean response among pixels of an image and is basically due to the dark signal non-uniformity (DSNU) and the photo response non-uniformity (PRNU). As mentioned above, the spatial non-uniformities of a sensor array are the same for every exposure; therefore the difference of two images removes this effect. The PTM uses this property to remove the effect of the spatial non-uniformity and thus to determine the parameters characterizing the sensor radiometry.

If temporal non-uniformity is present in the behaviour of a sensor, that is, the mean response is not stationary with respect to time, then the temporal noise does not represent the different photo-response among pixels. Therefore, the computed parameters by the PTM are meaningless.

There also exist patterns or periodic spatial variations due to the optical system (shading and vignetting effects), manufacturing imperfections and electronic interferences, causing non-stationary and non-white signal in space. To detect and correct the periodic variations, the standards propose the computation of the spectrogram. Finally, the standards propose plotting the logarithmic and accumulated histograms to detect defect pixels (outliers).

## 3. Justification of Our Approach

First of all, the procedure developed in this paper is not intended to replace any standard. Basically, the approach deeps in the grey level output signal (instead of physical radiometric properties) that interests professionals and users of digital cameras. Furthermore, an exploratory point of view is presented to analyze the noise of the image sensor reporting total quantities of noise according to the experimental factors of practical imaging processes. The strength is the flexibility of the linear models formulation which allows the noise to be explained and reported according to factors such as reflectance, wavelength, time, space and others.

The only requirement that the sensor must satisfy for the application of the DoE for LM is to provide independent and identically distributed measurements. Therefore, each pixel measurement is an independent realization of the random variable (grey level).

We are interested in quantifying the noise of the grey values according to the reflectance, wavelength and time factors. Furthermore, the noise is analyzed considering just a single image and a sequence of images.

Following the standards described in Section 2.1, PTM can be used to characterize sensor radiometry, quantum efficiency, system gain, saturation signal, *etc.* Furthermore, if the assumptions presented the standards are not fulfilled, the noise quantification and the noise components defined by our procedure still remain completely meaningful and useful. The approach developed in this paper decomposes the analysis of the uncertainty due to the sensor system into the noise present on a single image, the noise present on a combination of several images, *i.e.*, a sequence of images, and the temporal trend effect present on a sequence of images. The noise has a random probability distribution and the temporal trend is a systematic effect.

It is worth noting that the combination of several images does not imply any arithmetical operation among images, it only implies to consider jointly the data of the images.

In this paper, the evaluation of the total quantities of noise present on both a single image and a sequence of images are presented, rather than the evaluation and decomposition of the total noise into its components: shot noise, dark noise, quantification noise, DSNU and PRNU; quantum efficiency, overall system gain, saturation signal, absolute sensitivity threshold, and other parameters characterizing the radiometry of a sensor can be computed following the standards.

The single image noise of our procedure (Section 8.2) includes the temporal noise and the fixed pattern noise described by the standards. Likewise, the image sequence noise (Section 8.3) includes the temporal noise and the fixed pattern noise described in the standards and, in addition, temporal non-uniformity random effects on the signal. It should be noted that the standard does not consider possible temporal non-uniformity on the signal, that is, the photo-response noise (temporal noise) is considered as stationary and white with respect to time, as mentioned in Section 2.1. If temporal non-uniformity random effects are present, then the fixed pattern noise and the temporal noise cannot be separated and, therefore the PTM cannot be applied [6]. We show that the mean of the noise is not invariant with respect to time exhibiting random non-uniformity. Furthermore, the procedure developed in this paper evaluates possible temporal trend effects (Section 8.4), in contrast to the standards.

## 4. Mathematical Model of the Imaging System

The digital imaging process is a spatial sampling of the electromagnetic radiation from an object scene into the camera. For each sampling unit (*x_i_, y_j_*) sensitive to a small part of the electromagnetic spectrum, the radiometric response value *F* (grey level value) is measured by a sensor element targeting a surface patch of the object scene during a specified integration time. The radiometric values can be measured at different times. Thus, the imaging process is a spatio-temporal sampling of radiometric values, Equation (1). Each sampling unit (*x_i_, y_i_*) is named *pixel* and is represented by integer coordinates at different times. The radiometric response value *F* is recorded as an integer number called *digital code value, intensity value or grey level value*:

(1) F(x,y,t)={F∈N:(x,y,t)∈N}

### 4.1. Electromagnetic Radiation Function

An electromagnetic radiation function *C* has a spatial distribution (*x, y*) on an image at a defined time (*t*) and is dependent on the wavelength *λ* of the incoming light. Therefore, function *C* can be modelled by four independent parameters [20,21], Equation (2). The electromagnetic radiation is a real, positive and finite quantity. The values are higher than cero because the absence of light is not contemplated. The imaging system also imposes some restriction on the maximum electromagnetic radiation values:

(2) 0<C(x,y,t,λ)<∞

The electromagnetic radiation can be absorbed, transmitted, or reflected by an object. We consider the reflected energy case. If *E*(*x, y, t, λ*) represents the energy emitted from some primary light source and *r*(*x, y, t, λ*) represents the reflectance of an object scene, the radiant energy reflected from an object scene can be modelled by:

(3) C(x,y,t,λ)=E(x,y,t,λ)⋅r(x,y,t,λ)

The nature of *E*(*x, y, t, λ*) is determined by the illumination source, just like *C*(*x, y, t, λ*):

(4) 0<E(x,y,t,λ)<∞

However, *r*(*x, y, t, λ*) is determined by the characteristics of the imaged objects and is defined on the opened interval [0,1] to avoid considering both null reflectance and total reflectance values:

(5) 0<r(x,y,t,λ)<1

### 4.2. Radiometric Response Function

The radiometric response of an image sensor element, at a certain spatial unit on the image (*x, y*) and at a certain temporal unit (*t*), *F*(*x, y, t*), is measured in terms of the instantaneous luminance of the light field [20,21] and is defined by:

(6) $$F(x,y,t)=\int_{\lambda }C(x,y,t,\lambda )\cdot S(\lambda )d\lambda =\int_{\lambda }E(x,y,t,\lambda )\cdot r(x,y,t,\lambda )\cdot S(\lambda )d\lambda$$

where *S*(*λ*) is the spectral response of the sensor element. Trichromatic sensors obtain three independent functions corresponding to the Red, Green and Blue regions of the visible electromagnetic spectrum (wavelength bands):

(7) $$F_{R}(x,y,t)=\int_{\lambda }E(x,y,t,\lambda )\cdot r(x,y,t,\lambda )\cdot S_{R}(\lambda )d\lambda$$

(8) $$F_{G}(x,y,t)=\int_{\lambda }E(x,y,t,\lambda )\cdot r(x,y,t,\lambda )\cdot S_{G}(\lambda )d\lambda$$

(9) $$F_{B}(x,y,t)=\int_{\lambda }E(x,y,t,\lambda )\cdot r(x,y,t,\lambda )\cdot S_{B}(\lambda )d\lambda$$

In an image, the original continuous spatial distribution (*x, y*) is sampled by homogeneous cells (image spatial units), called pixels. For each pixel in the image, the radiometric response is measured by an independent sensor element [22].

The radiometric response involves two processes: image sensing and quantization. The former refers to converting the electromagnetic energy incident at the sensor element to a proportional output electrical signal and the latter to transforming the output electrical signal generated by each sensor element into a digital code value. Therefore, *F*(*x, y, t*) is an integer, positive and finite quantity named grey level or radiometric value:

(10) 0<F(x,y,t)<∞

Equation (10) represents the radiometric response process of the sensors and can be defined in the spatial (*x, y*) and temporal (*t*) dimensions.

## 5. Brief Description of the Approach

The developed procedure models and characterizes the radiometric response (grey level values) as well as the noise of a single image and the noise and trend of a temporal sequence of images due to the sensor system of a camera.

The procedure is entirely based on statistical and experimental techniques, in particular, Design of Experiments (DoE) for Linear Models (LM) [23–25]. The procedure is to formulate and adjust a LM to explain the radiometric response function according to all influential factors Equation (6) and to analyze the variance components of the model. Next, the resulting residual variance component and the variance component associated to the sequential factor are analyzed by formulating new LMs also according to the influential factors, to obtain the characterization of the noise and temporal trend of the sensor.

The spatial non-uniformity due to the sensor system (Table 1) is considered distributed randomly across the sensor array, therefore it is determined by an independent parameter of the coordinates (*x, y*). The uncertainty effects associated to the spatial factors (*x, y*) is minimized using a optimum optical system, with spatial uniform illumination and fixing shooting conditions. Therefore the spatial factors (*x, y*) are not considered in the model.

The uncertainty sources of scene changes in illumination or reflectance, geometric shooting conditions, integration bidirectional reflectance of the objects, time integration and additional imaging operators (Table 1) are also factors affecting the imaging process. However, the effects of these factors are minimized in our experiments. Therefore they are not considered in our model.

The sequential factor *t* is an extrinsic factor of the radiometric response function, Equation (1). Furthermore, the radiometric response function also depends on other factors: the primary light radiation *E*, the reflectance *r* and the spectral response *S*(*λ*) Equation (6). All these influential factors have to be taken into account in the formulation of the LM. Also, the minimization of the undesirable effects mentioned in the previous paragraphs must be carried out in the experimentation. In this context, the residual variance component (residual variability) of the model is an estimate of the sensor noise of the sequence of images (composed of the noise of a single image and the temporal non-uniformity random effects due to the sequential factor *t*) and the temporal variance component associated to the sequential factor *t* is an estimate of the sensor temporal trend.

## 6. Grey Level and Noise Modelling

**Scheme 1.**
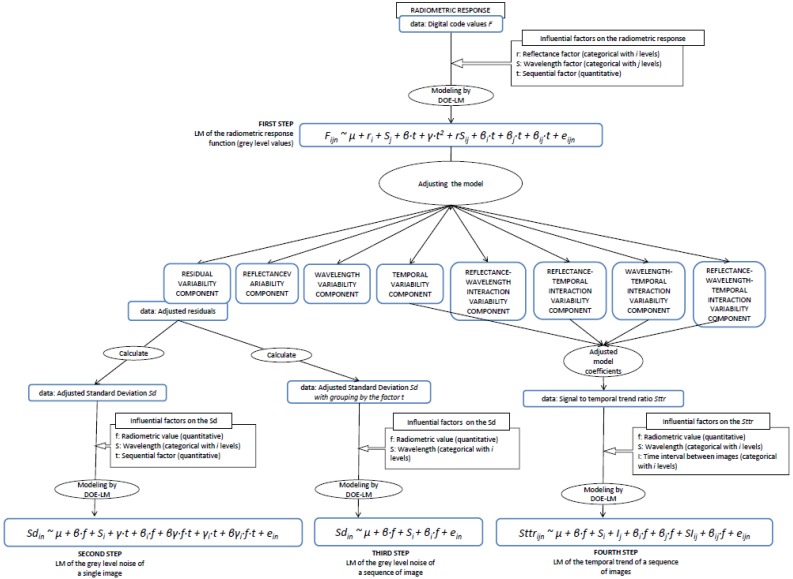
Scheme of the approach developed in this paper to analyze the radiometric values (grey level values), the sensor noise of a single image, the sensor noise of a sequence of images and the sensor temporal trend of a sequence of images.

The theoretical and experimental approach developed to analyze the radiometric values, the noise of a single image and the noise and trend of a temporal sequence of images consists of four steps. The procedure is depicted in Scheme 1 and allows us to establish the sequence of steps followed to evaluate the sensor.

The first step is to characterize the radiometric values. The radiometric response Equation (6) is modelled by DoE for LM (Section 6.1), adjusted by least squares and analyzed by means of the resulting Analysis of Variance (ANOVA) table, the adjusted model coefficients and the interaction plots (Section 8.1). The linear model relates the radiometric values (grey levels in raw units) to the influential factors (*x, y, t, E, r* and *S*(*λ*) in Equation (6)). The residual variance component of the model is the random variability (noise) of the process, composed of both the noise of a single image and the temporal non-uniformity random effects due to the sequential factor. The trend effect due to the sequential factor is represented by the temporal variance component of the model.

The second step is to characterize the sensor noise of a single image. A new linear model relating the adjusted residuals (resulting from the first step) to the influential factors is formulated (Section 6.2) and analyzed (Section 8.2), resulting in the quantification of the sensor noise of a single image according to the influential factors.

The third step models the adjusted residuals according to the influential factors except the sequential factor *t*, since the data has been joined by this factor, obtaining thus the quantification of the noise present in a combination of several images (sequence of images) (Sections 6.3 and 8.3).

The fourth step is to characterize the temporal trend effect due to the sensor nonstationary of the sensor in a sequence of images. The signal to temporal trend ratio (*Sttr*) is calculated by the adjusted model coefficients resulting from the radiometric response model (first step). This ratio indicates the proportion of signal temporally degraded until its stabilization. To characterize the temporal trend effect according to the influential factors, a new linear model to explain the *Sttr* is formulated (Section 6.4) and analyzed (Section 8.4). For this analysis we generated new sequences of images with different time intervals. The time interval *I* between images was included as a new categorical factor in the model.

### 6.1. Modelling the Radiometric Response Function

A linear model (LM) relating the radiometric values (grey level values) to a linear combination of all factors that affect the radiometric response is formulated. Equation (6) is the mathematical model of the process *F*(*x, y, t*) and it is dependent on three intrinsic factors, namely the primary light radiation *E*, the reflectance *r* and the spectral response *S*(*λ*); and also of three extrinsic factors, namely the spatial factors (*x, y*) and sequential factor *t*.

In this study, the light source *E* is considered constant by fixing the geometric frame and the illuminant, therefore it is not considered in the model. As mentioned above, the spatial non-uniformity effect is considered distributed randomly across the image and the rest of sources of spatial variability (Table 1) are minimized in our experiments. Therefore, the spatial factors (*x, y*) are not considered in the model.

The spectral response factor *S*(*λ*) determines the observed electromagnetic spectrum range. If the sensor elements are trichromatic, they obtain the radiometric response of the three independent red, green and blue bands of the visible electromagnetic spectrum. Therefore, the spectral response factor *S*(*λ*) is considered in the model by a categorical factor of fixed effects with three levels denoted by R, G and B.

The reflectance factor *r* is the principal factor influencing the radiometric response function. Note in Equation (6) that, for a particular electromagnetic band, the radiometric values are linearly proportional to the reflectance factor. Therefore, the number of sampled values (levels) of the factor *r* is not critical in the analysis and a sparse sampling covering almost all its range suffices.

The reflectance factor *r* is a physical characteristic of the scene objects and is defined on the continuous open interval [0,1] (Equation (5)). Since these true reflectance values are unknown, we model this factor as a categorical factor of fixed effects.

The sequential factor *t* causes a trend effect which is considered as a covariate in the model through a quantitative factor with quadratic component to embraces a possible curve effect. Finally, the residual factor *e* is also considered in the model. The expression of the LM for the radiometric response function is shown in Equation (11). The interactions with the quadratic component of the quantitative factor *t* are not considered in the model because are negligible effects:

(11) $$F_{\text{ijn}}∼\mu +r_{i}+S_{j}+\beta \cdot t+\gamma \cdot t^{2}+rS_{ij}+\beta_{i}\cdot t+\beta_{j}\cdot t+\beta_{ij}\cdot t+e_{\text{ijn}}$$

where:
*F_ijn_*n^th^-observation of the radiometric value *F* within the i^th^-level of the factor *r* and the j^th^-level of the factor *S*(*λ*)*μ*general average of *F**r_i_*effect of the i^th^-level of the reflectance factor *r**S_j_*effect of the j^th^-level of the spectral response factor *S*(*λ*)*t*value of the sequential factor *t* covariate for the *F_ijn_* observation*β*regression coefficient relating *F_ijn_* with the covariate *t**γ*regression coefficient relating *F_ijn_* with the covariate *t^2^**rS_ij_*interaction effect between the *r* and *S* factors*β_i_·t*interaction effect between the *r* factor and the covariate *t**β_j_·t*interaction effect between the *r* factor and the covariate *t**β_ij_·t*interaction effect among the *r, S* and the covariate *t**e_ijn_*residual of the *F_ijn_* observation

The whole uncertainty sources due to the optical camera system, the changing external conditions, the changing geometric shooting conditions, the bidirectional reflectance of the objects, the time integration and the additional imaging operators (Table 1) are also factors that might affect the imaging process. Nevertheless, these factors are minimized in our experimentation and are not considered in the model.

### 6.2. Modelling the Sensor Noise of a Single Image

The accurate characterization of the sensor noise of a single image is carried out with a new linear model relating the adjusted residuals resulting from the previous radiometric response model (Section 6.1) to the influential factors *r, S*(*λ*) and *t*. This model is formulated by introducing the adjusted standard deviation *Sd* instead of the adjusted residuals. Thus, the analysis and quantification are evaluated from the standard deviation indicator.

Furthermore, the adjusted radiometric value *F* resulting from the previous radiometric response model is used as an influential factor instead of the reflectance factor *r*, since both are linearly proportional, and the use of an adjusted radiometric value factor *F* is more interesting to characterize the noise. The adjusted radiometric values factor *F* is considered as a covariate in the model, through a quantitative factor *f* with linear trend effect. The expression of the sensor noise model is shown in Equation (12):

(12) $$Sd_{in}∼\mu +\beta \cdot f+S_{i}+\gamma \cdot t+\beta_{i}\cdot f+\beta \gamma \cdot f\cdot t+\gamma_{i}\cdot t+\beta \gamma_{i}\cdot f\cdot t+e_{in}$$

where:
*Sd_in_*n^th^-observation of the residual standard deviation *Sd* within the i^th^-level of the factor *S*(*λ*)*μ*general average of *Sd**S_i_*effect of the i^th^-level of the wavelength factor *S*(*λ*)*f*value of the radiometric value *F* covariate for the *Sd_in_* observation*β*regression coefficient relating *Sd_in_* with the covariate *f**t*value of the sequential factor *t* covariate for the *Sd_in_* observation*γ*regression coefficient relating *Sd_in_* with the covariate *t**β_i_·f*interaction effect between the *S* factor and the covariate *f**βγ·f·t*interaction effect between the covariate *f* and the covariate *t**γ_i_·t*interaction effect between the *S* factor and the covariate *t**βγ_i_·f·t*interaction effect among the covariate *f*, the covariate *t* and the factor *S**e_in_*residual of the *Sd_in_* observation

### 6.3. Modelling the Sensor Noise of a Sequence of Images

The linear model relates the residual adjusted standard deviation *Sd* resulting from the radiometric response model (Section 6.1) to the influential factors *F* and *S*(*λ*). The *Sd* is calculated by joining the data by the factor *t*, consequently this analysis represents the noise of a combination of several images (sequence of images), which is composed of the noise of a single image plus the temporal non-uniformity random effect.

The adjusted radiometric value factor *F* is considered as a covariate in the model, through a quantitative factor *f* with linear trend effect. The expression of the sensor noise model is shown in Equation (13):

(13) $$Sd_{in}∼\mu +\beta \cdot f+S_{i}+\beta_{i}\cdot f+e_{in}$$

where:
*Sd_in_*n^th^-observation of the residual standard deviation *Sd* within the i^th^-level of the factor *S*(*λ*)*μ*general average of *Sd**S_i_*effect of the i^th^-level of the wavelength factor *S*(*λ*)*f*value of the radiometric value *F* covariate for the *Sd_in_* observation*β*regression coefficient relating *Sd_in_* with the covariate *f**β_i_·f*interaction effect between the *S* factor and the covariate *f**e_in_*residual of the *Sd_in_* observation

### 6.4. Modelling the Temporal Sensor Trend

The temporal sensor trend effect can be quantified by means of the adjusted model coefficients resulting from the radiometric response model (Section 6.1). The *Sttr* indicates, for a certain radiometric value, the proportion of signal temporally degraded until its stabilization. To characterize the temporal trend effect according to the influential factors, a new linear model is formulated.

The time interval between images can be a relevant factor on the *Sttr*, therefore new sequences of images with different time intervals have to be acquired and the time interval *I* must be included as a new categorical factor in the model:

(14) $${\text{Sttr}}_{\text{ijn}}∼\mu +\beta \cdot f+S_{i}+I_{j}+\beta_{i}\cdot f+\beta_{j}\cdot f+SI_{ij}+\beta_{ij}\cdot f+e_{\text{ijn}}$$

where:
*Sttr_ijn_*n^th^-observation of the *Sttr* within the i^th^-level of the factor *S*(*λ*) and the j^th^-level of the factor *I**μ*general average of *Sttr**S_i_*effect of the i^th^-level of the spectral response factor *S*(*λ*)*I_j_*effect of the j^th^-level of the time interval factor *I**f*value of the radiometric value *F* covariate for the *Sttr_jn_* observation*β*regression coefficient relating *Sttr_ijn_* with the covariate *f**β_i_·f*interaction effect between the *S* factor and the covariate *f**β_j_·f*interaction effect between the *I* factor and the covariate *f**SI_ij_*interaction effect between the *S* and *I* factors*β_ij_·t*interaction effect among the *S, I* and the covariate *t**e_ijn_*residual of the *Sttr_ijn_* observation

## 7. Data Acquisition

The performed DoE is based on a crossover design, therefore *n* (*n* ≥ *1*) measurements of radiometric value for each combination of levels and factors are required.

As mentioned in Section 4.2, each pixel is a realization of the radiometric variable of a sensor element. If the sensor is trichromatic, there will be three radiometric values for the red, green and blue bands.

**Figure 1.**
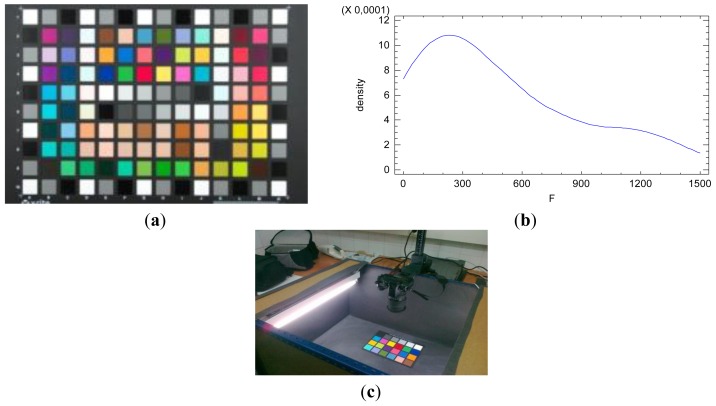
(**a**) Colorcheker chart formed by 140 regions with different reflectance characteristics; (**b**) Probability function of the radiometric values *F*; (**c**) Experimental light booth.

Furthermore, by imaging a colorcheker we can obtain the radiometric response for different reflectance values, since it is made of different regions with different reflectance characteristic, Figure 1(a). The colorcheker is a representative sample of reflectance values existing in real objects.

The reflectance factor is a physical characteristic of the scene objects and is defined on the continuous open interval [0,1] (Equation (5)). Since the true reflectance value of each one of the reflectance patch within the colorcheker is unknown, this factor is considered as a categorical factor of fixed effects in the model. The distribution function of the radiometric response values imaging the colorcheker is shown in Figure 1(b). It is confirmed the non-normal distribution of the radiometric values *F*.

The colorcheker was sequentially shot, resulting in *n* = *60* measurements, one per pixel, for each reflectance patch and at different times. Each measurement provides three colour components without interpolation, one for each band, due to the three layer structure of the Foveon X3 image sensor.

An example of the arranged data structure after observation is shown in Table 2. The *F* column contains the measured radiometric values in raw digital numbers ranging from 0 to 2^12^-1. The *id* column is the index of each pixel within the reflectance patch (level of the reflectance factor *r*—categorical factor). The *r* column is the level of the reflectance factor *r*. The *S* column is the level of the spectral response factor *S*(*λ*) (wavelength band) and *t* is the temporal sequence.

The independency and randomness hypothesis of the observations are guaranteed because, as mentioned above, each pixel corresponds with a realization of the radiometric variable by a sensor element and all of them are considered equal and independent [22].

## 8. Grey Level and Noise Analysis

Four corresponding linear models are formulated for the radiometric values, the sensor noise of a single image, the sensor noise of a sequence of images and the sensor temporal trend of a sequence of images. The four linear models will be adjusted by least squares and analyzed by the resulting ANOVA table, the adjusted model coefficients and the interaction plots.

### 8.1. Analysis of the Radiometric Response Function Model

The model was adjusted using 60 (*n*) observations of radiometric values for each combination of levels and factors. The factor *r* was sampled by 12 different levels covering the whole range of the colorcheker reflectance values. The sequence of images covers a period of 70 min, with intervals of 15 s between images. The sequential factor *t* is sampled by 80 regular gaps along the whole sequence. Finally, the spectral response factor *S*(*λ*) is experimented for the red (R), green (G) and blue (B) bands.

**Figure 2.**
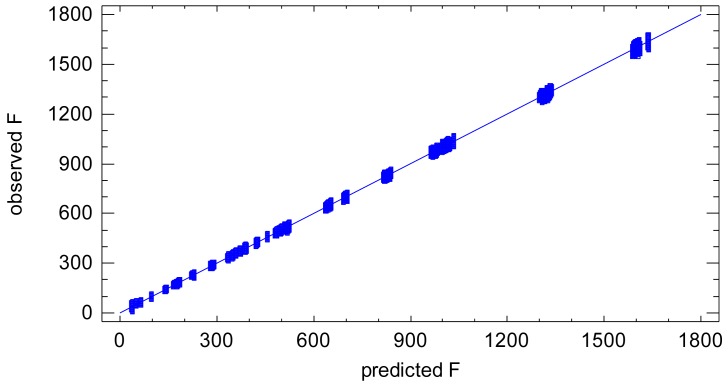
Goodness-of-fit graphic of the lineal model. Observed *vs.* adjusted radiometric values. It can be observed the linearity of the process. The separation with respect the straight line is the error or residual.

The resulting ANOVA table is shown in Table 3. All the effects included in the model are largely statistically significant (see P-Value column in the ANOVA table). The model has a goodness-of-fit of 99.97% (R-square in ANOVA table), Figure 2. This suggests that under uniform external conditions the imaging process can be modelled very accurately by a linear model. The variability of the model, *i.e.*, residual variability, equals to the 0.03% of the total variability. Since all the influential factors affecting the experimental process are included in the model, the residual variability can be assigned to the sensor noise of the process of a sequence of images, which is composed of both the noise of a single image and the temporal non-uniformity random effects.

**Figure 3.**
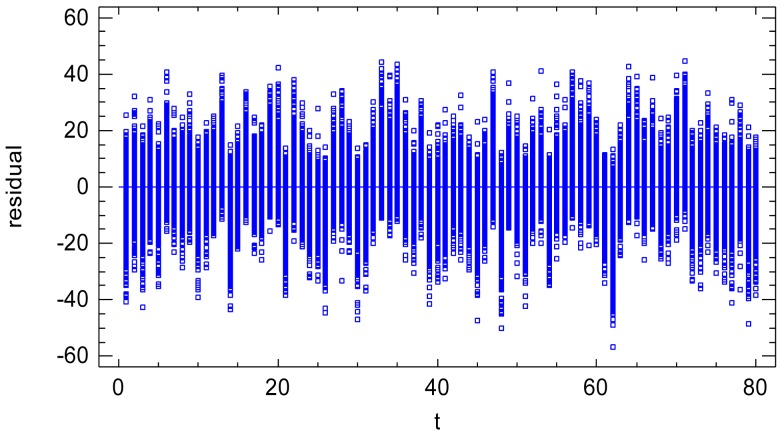
Residuals *vs.* sequential factor *t*.

The validation plot of residual *vs.* sequential factor, Figure 3, shows that the sequential factor *t* does not cause heteroscedasticity [23,24] on the residuals. However, it has a small random effect on the residual mean value. Therefore, it is confirmed that the residual variance component is composed of both the noise of a single image (variance at certain time) and the temporal non-uniformity random effects. The latter will be quantified in Section 8.3.

The statistical significance tests in the ANOVA table are based on two basic hypotheses: the normality of the conditional distribution of the residuals and the homogeneity of the residual variance, *i.e.*, homoscedasticity of the process. These two hypotheses are tested by means of the validation plots.

**Figure 4.**
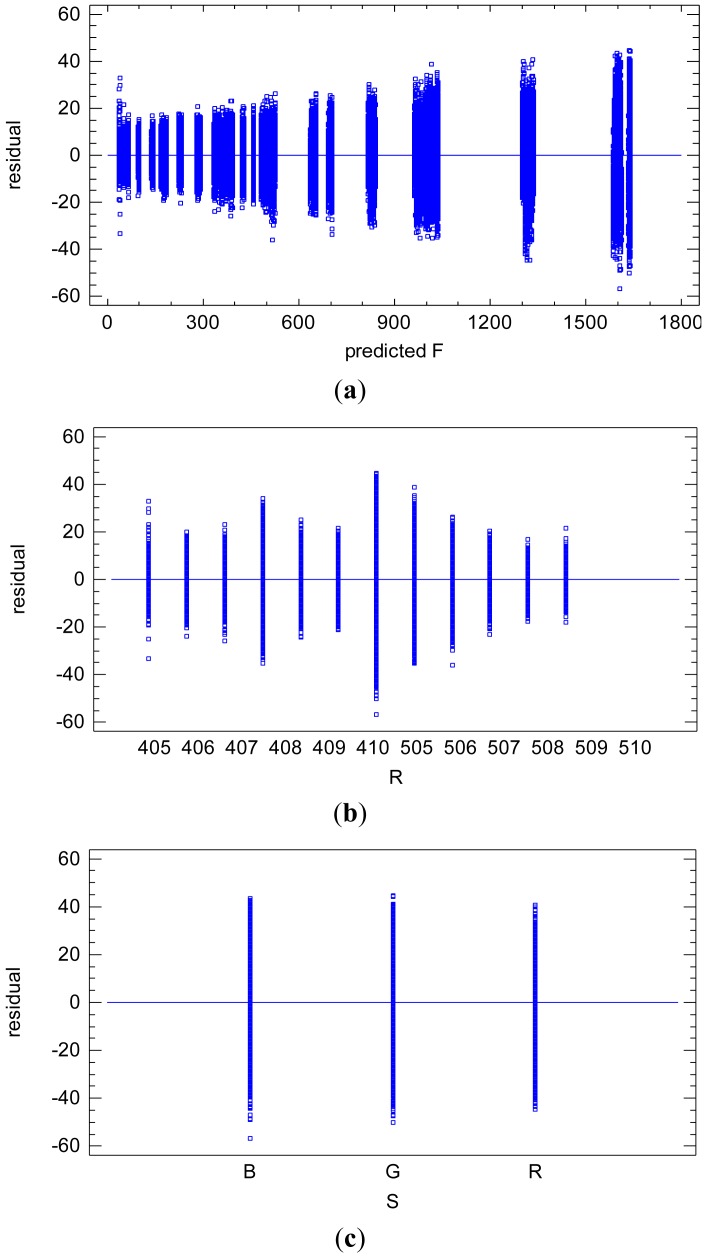
(**a**) Residuals *vs.* adjusted radiometric values *F* illustrating the heterogeneity of the residuals; (**b**) Residuals *vs.* reflectance factor *r* (Note that factor *r* is a categorical factor); (**c**) Residuals *vs.* wavelength factor *S*.

Figure 4(a) shows the heterogeneity of the residual variance. The residuals are linearly related with the adjusted radiometric values *F*, increasing accordingly. It is clear that the heterogeneity of the residuals is also caused by the reflectance factor *r*, Figure 4(b). Note the linear proportional relation between the radiometric value *F* and the reflectance factor *r*, Equation (6). The spectral response factor *S*(*λ*) does not cause significant heterogeneity on the residuals, Figure 4(c).

The slight deviation from the homoscedasticity hypothesis does not invalidate the significant tests (*F-Ratio* and *P-Value*) in the ANOVA table due to the unequivocal significance of them.

**Figure 5.**
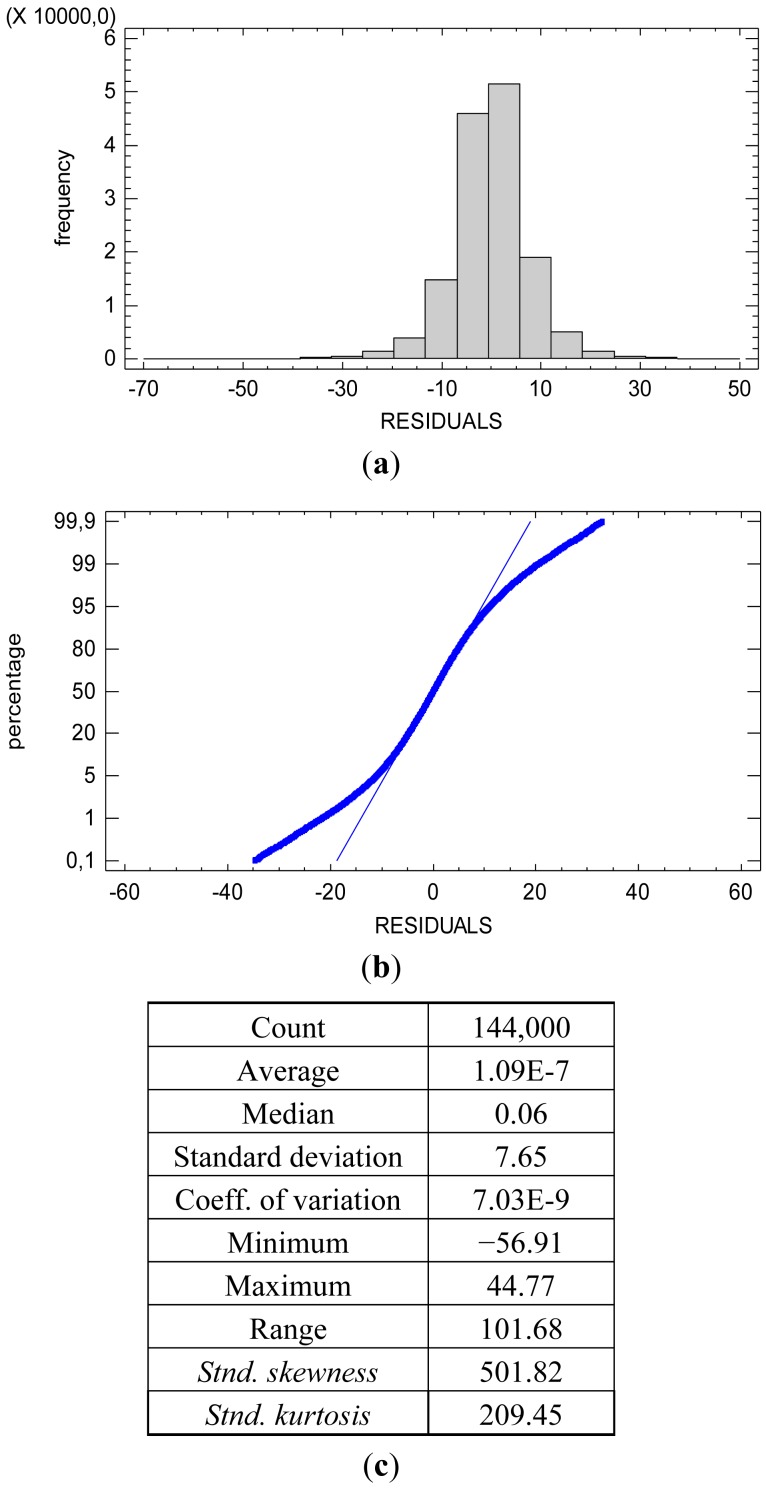
Descriptive statistics and plots for the totality of the residuals. (**a**) The histogram of the residuals; (**b**) The normal probability plot for the residuals illustrates the slight failing of the normal distribution; (**c**) The skewness and kurtosis coefficients indicate the slight fail of the normal distribution.

**Figure 6.**
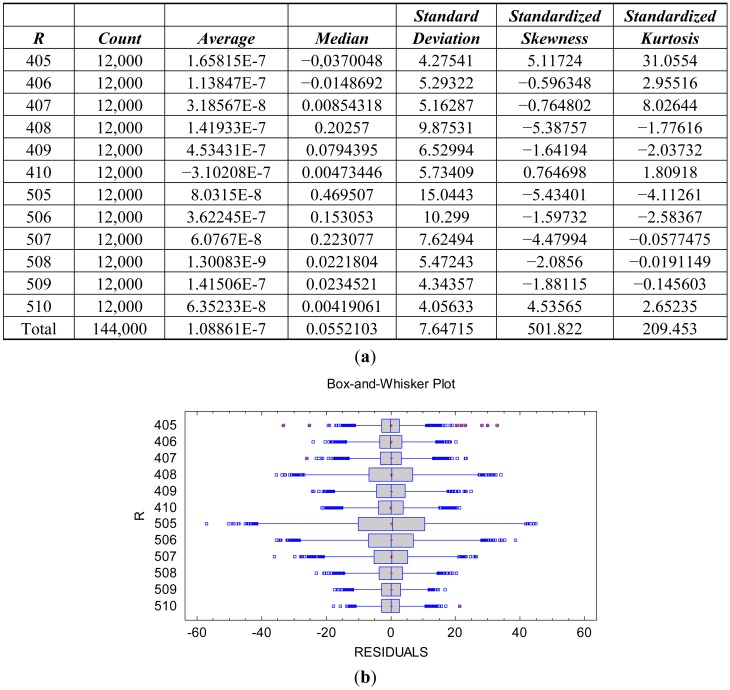
Descriptive statistics (**a**) and plots (**b**) for the residuals of the wavelength R (red) for the 12 levels of the reflectance factor.

The heterogeneity of the residual variance causes the slight non-normality of the distribution for the totality of the residuals, Figure 5. The hypothesis of the significance tests in the ANOVA table is related to the normality of the conditional distribution of the residuals. The distribution of the residuals conditioned to both the reflectance factor *r* and the spectral response factor *S*(*λ*) is normal. Figure 6 shows the descriptive statistics and plots confirming the normality of the conditional distribution for the spectral response factor *S*(*λ*) with *λ* = *R* (red). The conditional distribution of the spectral response factor *S*(*λ*) with *λ* = *G* (green) and with *λ* = *B* (blue) are also normal.

The heterogeneity of the residual variance makes the standard error statistic and the mean absolute error statistic in Table 3 approximate measures of the variability of the residuals and the average absolute value of the residuals, respectively. Furthermore, these two statistics are also conditioned to the experimented-sample of the reflectance factor *r*.

### 8.2. Analysis of the Sensor Noise of a Single Image Model

The accurate characterization of the sensor noise of a single image is carried out with a new linear model to explain the adjusted standard deviations *Sd* according to the influential factors *f, S*(*λ*) and *t*. The *Sd* was calculated through the adjusted residual variability of the radiometric response model (Section 8.1).

The resulting ANOVA table is shown in Table 4. The effect of the factor *t* and its interaction effect with the rest of the factors are not statistically significant (P-Value in the ANOVA table). It is confirmed that the sequential factor *t* does not influence the standard deviation indicator *Sd*. Consequently, and because the standard deviation indicator *Sd* has been calculated independently for each level of the sequential factor, this analysis represents the sensor noise of a single image.

Eliminating the factor *t* of the model and adjusting the model again, a new resulting ANOVA table is obtained in Table 5. The model has a goodness-of-fit of 88.07% (R-square in ANOVA table). The standard error statistic is 0.57 raw units which is a measure of the residual variability of the adjustment. The mean absolute error is 0.45 raw units which is the mean absolute value of the residuals of the adjustment.

**Figure 7.**
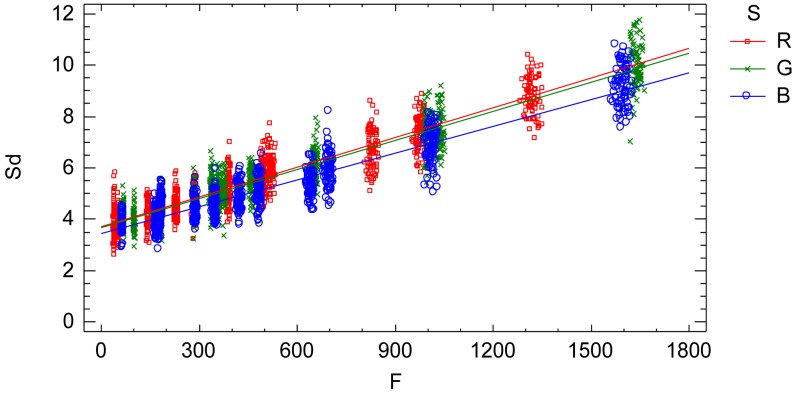
Interaction plot. Standard deviation *vs.* adjusted radiometric values *F* depending on the wavelength factor. It is the characterization of the sensor noise (standard deviation) of a single image according to the influential factors.

The interaction plot from the adjusted model, Figure 7, characterizes and quantifies the standard deviation *Sd* which is an indicator of the sensor noise. The influential factors are the radiometric value factor *f* and the spectral response factor *S*(*λ*). Thus, the standard deviation of the process of a single image is characterized by a normal distribution with zero mean and variance Equation (16) depending on the radiometric value and the electromagnetic band (Figure 7 and Equations (17), (18) and (19)):

(16) $$e_{f,S}\sim N(0,Sd_{f,S}^{2})$$

(17) $$Sd_{f,S=R}=3.70796+0.0038508f$$

(18) $$Sd_{f,S=B}=3.66072+0.0037864f$$

(19) $$Sd_{f,S=B}=3.42795+0.0034809f$$

**Figure 8.**
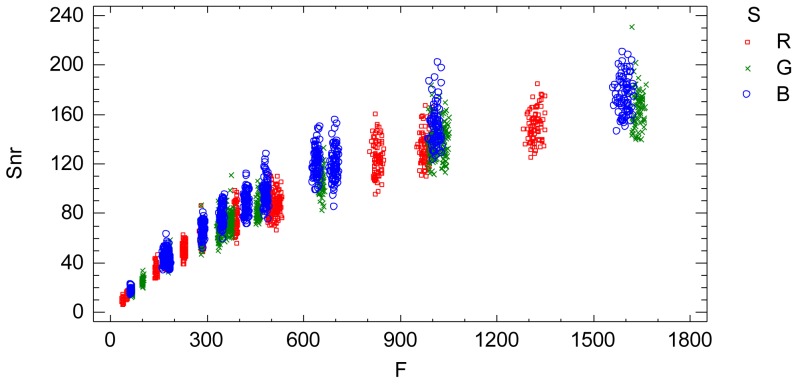
Signal to noise ratio for the three level of the wavelength factor: R (red colour), G (green colour) and B (blue colour).

The signal-noise ratio (*Snr*) is an indicator of the relative proportion of noise contained in the signal (radiometric value), Figure 8. Large values of *Snr* indicate low noise in the signal and vice versa.

### 8.3. Analysis of the Sensor Noise of a Sequence of Images

The linear model relates the residual adjusted standard deviation *Sd* resulting from the radiometric response model (Section 8.1) to the influential factors *f* and *S*(*λ*). The *Sd* is calculated joining the data by the factor *t*, consequently this analysis represents the noise of the sequence of images (noise of a single image plus the temporal non-uniformity random effect).

The resulting ANOVA table is shown in Table 6. The standard error statistic is 0.50 raw units and the mean absolute error is 0.39 raw units. The model has a goodness-of-fit of 97.17% (R-square in ANOVA table).

**Figure 9.**
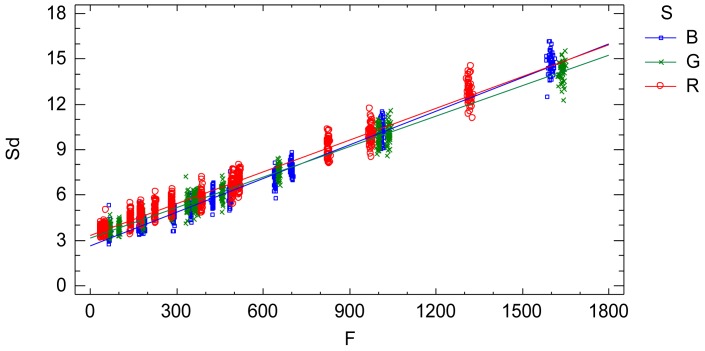
Interaction plot. Standard deviation *Sd vs.* adjusted radiometric values *F* depending on the wavelength factor. It is the characterization of the sensor noise (standard deviation) of a sequence of images according to the influential factors.

The interaction plots from the adjusted model, Figure 9, characterizes and quantifies the standard deviation *Sd* which is an indicator of the sensor noise. The influential factors are the radiometric value factor *f* and the spectral response factor *S*(*λ*). Thus, the standard deviation of the process of a sequence of images is characterized by a normal distribution with zero mean and standard deviation Equation (20) depending on the radiometric value and the electromagnetic band according to Figure 9 and Equations (21), (22) and (23):

(20) $$e_{f,S}\sim N(0,Sd_{f,S}^{2})$$

(21) $$Sd_{f,S=R}=3.3175+0.00699187\cdot f$$

(22) $$Sd_{f,S=G}=3.1735+0.00670485\cdot f$$

(23) $$Sd_{f,S=B}=2.6629+0.00738875\cdot f$$

**Figure 10.**
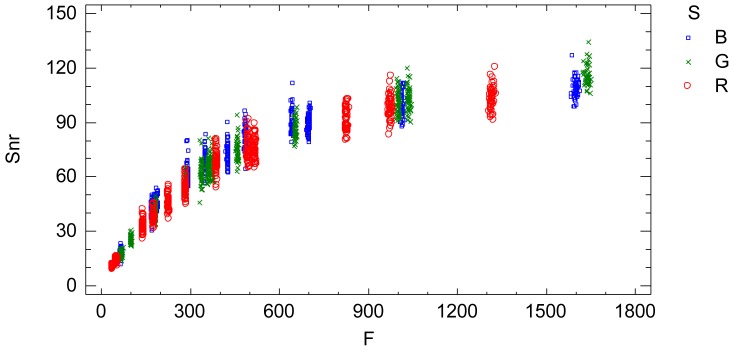
Signal to noise ratio for the three level of the wavelength factor: R (red color), G (green color) and B (blue color).

The signal to noise ratio (*Snr*) is an indicator of the relative proportion of noise contained in the signal (radiometric value), Figure 10. Large values of *Snr* indicate low noise in the signal and vice versa.

### 8.4. Analysis of the Sensor Temporal Trend

**Figure 11.**
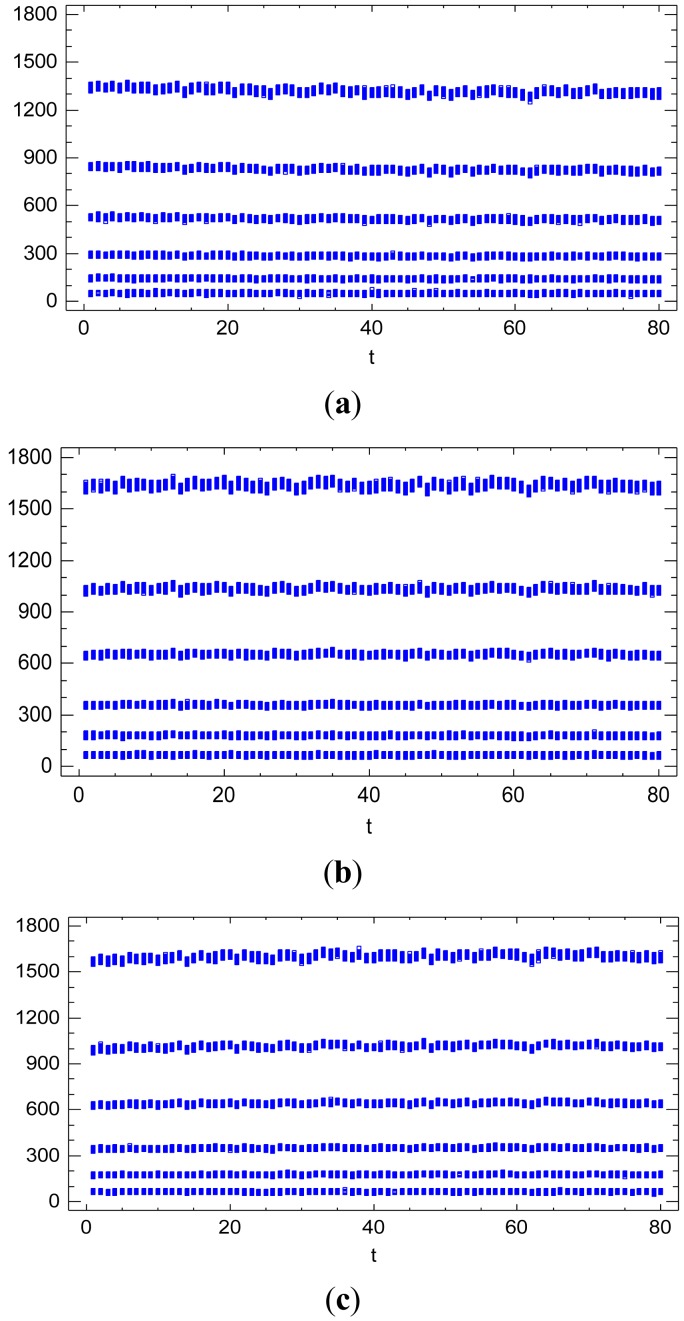
Temporal evolution of the radiometric values in the sequence of images. Evolution for the spectral response factor *S*(*λ*) with *λ* = *R* (**a**). With *λ* = *G* (**b**). With *λ* = *B* (**c**).

Figure 11 illustrates the temporal trend effect due to the sequential factor *t* on the radiometric values for each band, *i.e.*, the spectral response factor *S*(*λ*). It can be observed a very slight logarithmic trend towards the stabilization of the radiometric values, with different logarithmic forms for each level of the spectral response factor *S*(*λ*). Consequently, the temporal trend effect is considered as a covariate in the radiometric response function model (Section 6.1.) through a quantitative factor with a quadratic trend effect in Equation (11).

The *Sttr* indicates the proportion of signal temporally degraded until its stabilization for a certain radiometric value. To characterize and quantify the temporal trend effect according to the influential factors, a new linear model to explain the *Sttr* is formulated and analyzed. For this analysis, new sequences of images can be generated with different time intervals, and the time interval *I* between images can be included as a new influential factor in the model. Three different sequences of images have been obtained with time intervals of 5, 15 and 60 s between images.

**Figure 12.**
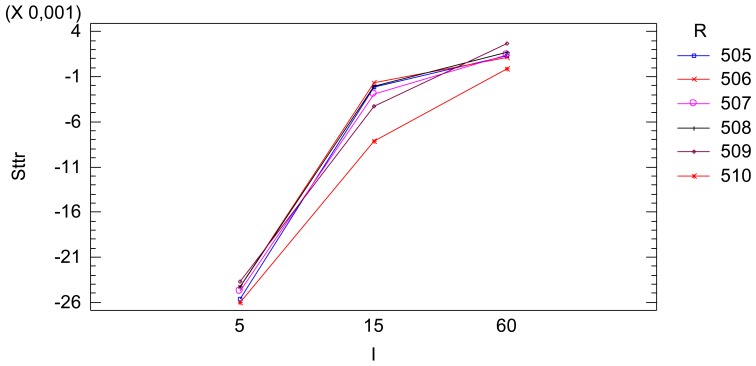
*Sttr vs.* time interval factor *I*, according to the reflectance factor *R* or radiometric value factor *F*, equivalently.

The resulting ANOVA table is shown in Table 7. The effect of the radiometric value factor *f* is not significant. This only has some significance when interacting with the time interval factor *I*. Eliminating the clear non-significant effects, that is, the effect of the interaction between the spectral response *S*(*λ*) and the radiometric values *f* and the effect of the triple interaction between spectral response *S*(*λ*), time interval *I* and the radiometric values *f*, and adjusting the model again, a new resulting ANOVA table is obtained (Table 8). The effect of the radiometric value factor *f* is not statistically significant, but its interaction effect with the time interval factor *I* has some significance. In the time interval of 15 s some variability due to the reflectance factor or radiometric value factor exists as can be seen in Figure 12. Nevertheless, this variability is quite small and we can consider that the radiometric value factor does not affect the *Sttr*.

**Figure 13.**
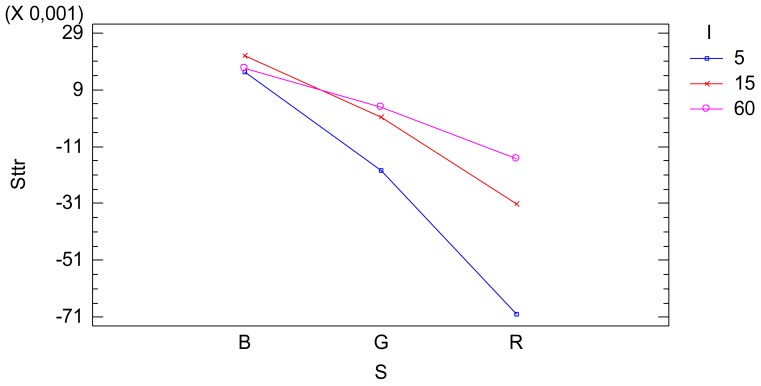
*Sttr vs.* spectral response factor *S*(*λ*), according to the 3 levels (5, 15, 60 s) of the time interval factor *I*.

The ultimate model relates the *Sttr* with the spectral response factor *S*(*λ*) and the time interval factor *I*, Table 9. The interaction plot, Figure 13, characterizes the temporal trend of a sequence of images, relating the *Sttr* to the spectral response *S*(*λ*) and the time interval *I* between images. The *Sttr* does not significantly depend on the radiometric value.

## 9. Discussion and Conclusions

The knowledge and the assessment of the behaviour of the grey level values registered by an image sensor as well as the uncertainty effects are essential keys to improve image processing applications, to finding out the limitations of the imaging systems and to calibrate imaging systems.

In this paper we performed the evaluation of an electro-optical sensor under laboratory conditions to characterize the grey level values on image sequences. The grey level value is modelled and characterized according to the influential factors in practical imaging processes. The grey level noise due to sensor system present in a single image and the grey level noise and temporal trend present in a sequence of images are analyzed. The noise is distributed randomly and the temporal trend is a systematic effect. The procedure is tested on a Foveon X3 image sensor featured in a Sigma SD15 digital SLR camera using raw units. The performance of the system can be carried out with standard laboratory equipment.

A practical and comprehensive characterization of the radiometric values (grey level values) is achieved under ideal laboratory conditions, that is, uniform lighting, controlled reflectance values and ideal shooting conditions. The characterization concerns the knowledge of the factors affecting the radiometric values under the specified laboratory conditions, namely the reflectance factor, the wavelength factor and the sequential factor. A linear model has been formulated to characterize the radiometric values achieving a goodness-of-fit of 99.97%, a standard error of 7.65 raw units and a mean absolute error of 5.45 raw units. The high goodness-of-fit percentage indicates that the linear model characterizes well the digital numbers under uniform external conditions, explaining the totality of the variability present in the radiometric response. The variability unexplained by the linear model, 0.03%, corresponds to the residual variability. Since all the factors affecting the performed experimental process have been modelled, this residual variability can be assigned to the noise of the sensor system. This residual variability is composed of both the noise of a single image and the temporal non-uniformity random effect due to the sequential factor. The standard error statistic is a measurement of the residual variability of the model and represents grey level noise due to the sensor system. But this statistic is a rough measure because the adjusted residuals are not homocedastic and it is also conditioned to the experimented sample of the reflectance factor *r*. The second objective is to obtain an accurate quantification of the grey level noise of the sensor system present in a single image according to the influential factors. In order to do this, a new model relating the residual variability to the influential factors (radiometric value, wavelength and sequence) has been formulated. The sensor noise is clearly related to the radiometric value, increasing with it, and slightly dependent on the wavelength (Figure 7). Since this model has been formulated to explain the adjusted standard deviation *Sd*, which has been calculated for each time interval independently, the results of this model represent the sensor noise of a single image. The signal to noise ratio is related to the radiometric values similarly to an increasing logarithmic function (Figure 8).

The third objective is to obtain an accurate quantification of the grey level noise of the sensor system present in a sequence of images according to the influential factors. The noise in a sequence of images is composed of the noise of a single image plus the temporal non-uniformity random effects. Therefore, the data has been joined by the sequential factor *t* and a model relating the residual variability to the radiometric value and the wavelength factors has been formulated. This analysis represents the noise of a sequence of images (Figure 9). The Foveon X3 image sensor has a low temporal non-uniformity random effect. Thus, it can be stated that the noise of a sequence of images is similar or slightly higher than the noise of a single image.

Finally, the sequential factor of a sequence of images has a trend effect on the radiometric values. This effect is similar to a logarithmic trend until the stabilization of the signal. The proportion of signal temporally degraded until its stabilization (*Sttr*) is a good indicator to characterize the temporal trend effect on the radiometric values. A new model relating the *Sttr* to the influential factors has been formulated. The *Sttr* is clearly related with the wavelength and the time interval between images of the sequence of images and it is not depended on the radiometric value. The *Sttr* is clearly significant with short time interval between images and it is negligible when the time interval is greater than 60 s. The wavelength corresponding to the red band has a greater *Sttr* and this is negative. The green band only has a significant *Sttr* when the time interval is very short and its *Sttr* is also negative. The blue band has a low *Sttr* equal for all time intervals and this is positive.

The temporal trend effect can be corrected on images since it is a systematic effect. However, the random effects, like the noise, cannot be corrected; they can only be taken into account in image processing applications.

The noise quantities computed in this paper are complementary to the standards ISO and EMVA. Whether the sensor does not exhibit temporal non-uniformity random effect, the PTM can be applied to compute the totality of the parameters characterizing the radiometry of a sensor. Furthermore, if the temporal non-uniformity random effect is present in an imaging process, the noise components and quantities analyzed in this paper are completely useful. The noise quantities are computed and reported according to the influential factor and continuously across all theirs ranges.

The presented approach is very flexible. It can be used to define different relations between the grey level values and the influential factors, and to model non-stationary behaviour regarding time and space, for example temporal trend, spatial trend (vignetting, shading and non-uniform illumination effects), in contrast to the standards. Also, the procedure is useful to model and analyze the effects of other factors such as the time integration, geometrical shooting conditions and so on. The unique condition that has to be satisfied in the application of our procedure is that each pixel provides independent and identically distributed measurements of the grey level value.

Future research will deal with the extension of the presented approach to model spatial variability effects such as vignetting and non-uniform illumination, as well as wavelength relationships.

## Figures and Tables

**Table 1.** Outline of the most important uncertainty sources and effects affecting the radiometric values in digital cameras. The uncertainty sources analyzed in this paper are highlighted.

**Components of Uncertainty**	**Uncertainty Sources And Effects**			**Appears When/In**
	**Sources**		**Effects**	
	**Camera**	**External conditions**		
Noise	**Sensor system**		Random	Single images
			Systematic	
	**Integration time**		Random	
			Systematic	
	**Additional imaging operators**		Systematic	
			Random	
		**Bidirectional reflectance of the object scene**	Random	
			Systematic	
Spatial Variability	**Sensor System**		Spatial non-uniformity	
	**Optical system**		Trend	
	**Sensor manufacturing imperfections**		Trend	
			Random	
		**Spatial non-uniformity illumination**	Systematic	
			Trend	
		**Geometric shooting conditions**	Trend	
Temporal Variability	**Sensor system**		Random (Temporal non-uniformity)	Comparing images at different times
			Trend	
		**Scene changes in illumination**	Trend	
		**Scene changes in reflectance**	Trend	

**Table 2.** Data structure example. (Note that the *r* and *S* factors are categorical).

***F* (*digital num. 2^12^*)**	***id***	***r***	***S***	***t***	***F* (*digital num. 2^12^*)**	***id***	***r***	***S***	***t***
660	1	101	R	1	895	2	101	B	1
661	2	101	R	1	‥	‥	101	B	1
‥	‥	101	R	1	‥	N	101	B	1
‥	N	101	R	1	800	1	102	B	1
231	1	102	R	1	797	2	102	B	1
230	2	102	R	1	‥	‥	102	B	1
‥	‥	102	R	1	‥	N	102	B	1
‥	N	102	R	1	‥	‥	‥	B	1
‥	‥	‥	R	1	‥	‥	R	B	1
‥	‥	R	R	1	900	1	101	R	2
265	1	101	G	1	895	2	101	R	2
263	2	101	G	1	‥	‥	101	R	2
‥	‥	101	G	1	‥	N	101	R	2
‥	N	101	G	1	800	1	102	R	2
502	1	102	G	1	797	2	102	R	2
506	2	102	G	1	‥	‥	102	R	2
‥	‥	102	G	1	‥	N	102	R	2
‥	N	102	G	1	‥	‥	‥	R	2
‥	‥	‥	G	1	‥	‥	R	R	2
‥	‥	R	G	1	‥	‥	‥	‥	‥
900	1	101	B	1	‥	‥	‥	‥	‥

**Table 3.** ANOVA table after adjusting the radiometric response function model. All the effects are statistically significant (P-Value). The standard error results 7.65 raw units. The mean absolute error results 5.57 raw units. The goodness-of-fit is 99.97%.

**General Linear Models**					
Number of dependent variables: 1					
Number of categorical factors: 2 (*R, S*)					
Number of quantitative factors: 2 (*t, t*^2^)					
**Sums of Squares**					

***Source***	***Sum of Squares***	***Df* (*degrees of freedom*)**	***Mean Square***	***F-Ratio***	***P-Value***

*R*	5.96068E9	11	5.4188E8	9,261,608.77	0.0000

*S*	3.74283E7	2	1.87142E7	319,855.19	0.0000

*t*	5,351.49	1	5,351.49	91.47	0.0000

*t*ˆ*2*	16,931.4	1	16,931.4	289.39	0.0000

R*S	1.96456E8	22	8.92981E6	152,624.99	0.0000

R*t	21,604.7	11	1,964.07	33.57	0.0000

S*t	902,480	2	451,240	7,712.42	0.0000

R*S*t	633.214	22	28,782.5	491.94	0.0000

Residual	8.42091E6	143,927	58.5082		

Total (corrected)	2.52327E10	143,999			

R-Squared = 99.97%					
R-Squared (adjusted for d.f.) = 99.97%					
Standard Error of Est. = 7.65					
Mean absolute error = 5.57					
Durbin-Watson statistic = 1.58 (P = 0.0000)					

**Table 4.** Resulting ANOVA table of the sensor noise model.

**General Linear Models**					
Number of dependent variables: 1					
Number of categorical factors: 1 (*S*)					
Number of quantitative factors: 2 (*f, t*)					
**Sums of Squares**					

***Source***	***Sum of Squares***	***Df***	***Mean Square***	***F-Ratio***	***P-Value***

*S*	2.25918	2	1.12959	3.42	0.0327

*f*	1,588.72	1	1,588.72	4,816.04	0.0000

*t*	0.298784	1	0.298784	0.91	0.3412

S*f	5.85899	2	2.9295	8.88	0.0001

f*t	1.15191	1	1.15191	3.49	0.0617

S*t	0.465308	2	0.232654	0.71	0.4941

S*f*t	0.924773	2	0.462387	1.40	0.2464

Residual	946.099	2,868	0.329881		

Total (corrected)	7,949.27	2,879			

R-Squared = 88.10%					
R-Squared (adjusted for d.f.) = 88.05%					
Standard Error of Est. = 0.57					
Mean absolute error = 0.45					
Durbin-Watson statistic = 2.04 (P = 0.1477)					

**Table 5.** Resulting ANOVA table of the sensor noise model without the effect of the sequential factor *t*.

**General Linear Models**					
Number of dependent variables: 1					
Number of categorical factors: 1 (*S*)					
Number of quantitative factors: 1 (*f*)					
**Sums of Squares**					

***Source***	***Sum of Squares***	***Df***	***Mean Square***	***F-Ratio***	***P-Value***

*S*	17.5761	2	8.78803	26.63	0.0000

*f*	6,771.23	1	6,771.23	20,516.05	0.0000

S*f	12.7137	2	6.35687	19.26	0.0000

Residual	948.551	2,874	0.330046		

Total (corrected)	7,949.27	2,879			

R-Squared = 88.07%					
R-Squared (adjusted for d.f.) = 88.05%t					
Standard Error of Est. = 0.58					
Mean absolute error = 0.45					
Durbin-Watson statistic = 2.04 (P = 0.1557)					

**Table 6.** Resulting ANOVA table of the image sequence sensor noise model for a Foveon X3 sensor (dynamic range 2^12^ raw units).

**General Linear Models**					
Number of dependent variables: 1					
Number of categorical factors: 1 (*S*)					
Number of quantitative factors: 2 (*f*)					
**Sums of Squares**					

***Source***	***Sum of Squares***	***Df***	***Mean Square***	***F-Ratio***	***P-Value***

*S*	57.7505	2	28.8752	114.64	0.0000

*f*	15,205.2	1	15,205.2	60,367.49	0.0000

S*f	26.2726	2	13.1363	52.15	0.0000

Residual	451.111	1,791	0.251877		

Total (corrected)	15,920.1	1,796			

R-Squared = 97.17%					
R-Squared (adjusted for d.f.) = 97.16%					
Standard Error of Est. = 0.50					
Mean absolute error = 0.39					
Durbin-Watson statistic = 1.77 (P = 0.0000)					

**Table 7.** Resulting ANOVA table of the temporal trend model.

**General Linear Model**					
Number of dependent variables: 1					
Number of categorical factors: 2 (*S, I*)					
Number of quantitative factors: 1 (*f*)					
**Sums of Squares**					

***Source***	***Sum of Squares***	***Df***	***Mean Square***	***F-Ratio***	***P-Value***

*S*	0.00860736	2	0.00430368	1,724.75	0.0000

*I*	0.00177015	2	0.000885074	354.70	0.0000

*f*	0.0000105471	1	0.0000105471	4.23	0.0471

S*I	0.00130896	4	0.000327241	131.15	0.0000

S*f	0.00000794286	2	0.00000397143	1.59	0.2176

I*f	0.0000257446	2	0.0000128723	5.16	0.0107

S*I*f	0.00000503574	4	0.00000125894	0.50	0.7326

Residual	0.000089829	36	0.00000249525		

Total (corrected)	0.0404397	53			

R-Squared = 99.78%					
R-Squared (adjusted for d.f.) = 99.67%					
Standard Error of Est. = 0.002					
Mean absolute error = 0.0010					
Durbin-Watson statistic = 2.44 (P = 0.3465)					

**Table 8.** Resulting ANOVA table of the temporal trend model without the interaction effects S*f and S*I*f.

**General Linear Models**					
Number of dependent variables: 1					
Number of categorical factors: 2 (*S, I*)					
Number of quantitative factors: 1 (*f*)					
**Sums of Squares**					

***Source***	***Sum of Squares***	***Df***	***Mean Square***	***F-Ratio***	***P-Value***

*S*	0.0284155	2	0.0142077	5,719.15	0.0000

*I*	0.00177372	2	0.00088686	356.99	0.0000

*f*	0.00000822607	1	0.00000822607	3.31	0.0759

S*I	0.0044165	4	0.00110412	444.45	0.0000

I*f	0.0000237051	2	0.0000118525	4.77	0.0136

Residual	0.000104338	42	0.00000248424		

Total (corrected)	0.0404397	53			

R-Squared = 99.74%					
R-Squared (adjusted for d.f.) = 99.67%					
Standard Error of Est. = 0.002					
Mean absolute error = 0.0010					
Durbin-Watson statistic = 2.25 (P = 0.3152)					

**Table 9.** Resulting ANOVA table of the temporal trend model, only with the effects of the spectral response factor *S*(*λ*) and the time interval factor *I*.

**General Linear Models**					
Number of dependent variables: 1					
Number of categorical factors: 2 (*S, I*)					
Number of quantitative factors: 0					
**Sums of Squares**					

***Source***	***Sum of Squares***	***Df***	***Mean Square***	***F-Ratio***	***P-Value***

*S*	0.0288969	2	0.0144484	4,496.89	0.0000

*I*	0.00694348	2	0.00347174	1,080.53	0.0000

S*I	0.00445472	4	0.00111368	346.62	0.0000

Residual	0.000144585	45	0.00000321299		

Total (corrected)	0.0404397	53			

R-Squared = 99.64%					
R-Squared (adjusted for d.f.) = 99.58%					
Standard Error of Est. = 0.002					
Mean absolute error = 0.001					
Durbin-Watson statistic = 1.79 (P = 0.0251)					
